# The Effect of Meteorological Variables on the Transmission of Hand, Foot and Mouth Disease in Four Major Cities of Shanxi Province, China: A Time Series Data Analysis (2009-2013)

**DOI:** 10.1371/journal.pntd.0003572

**Published:** 2015-03-05

**Authors:** Junni Wei, Alana Hansen, Qiyong Liu, Yehuan Sun, Phil Weinstein, Peng Bi

**Affiliations:** 1 Department of Epidemiology, School of Public Health, Shanxi Medical University, Taiyuan, Shanxi, China; 2 Discipline of Public Health, School of Population Health, The University of Adelaide, Adelaide, Australia; 3 State Key Laboratory for Infectious Diseases Prevention and Control, National Institute for Communicable Disease Control and Prevention, Chinese Center for Disease Control and Prevention, Beijing, China; 4 Shandong University Climate Change and Health Center, Jinan, Shandong, China; 5 Department of Epidemiology and Biostatistics, School of Public Health, Anhui Medical University, Hefei, Anhui, China; 6 Division of Health Sciences, School of Pharmacy and Medical Sciences, The University of South Australia, Adelaide, Australia; Genesis Laboratories, UNITED STATES

## Abstract

Increased incidence of hand, foot and mouth disease (HFMD) has been recognized as a critical challenge to communicable disease control and public health response. This study aimed to quantify the association between climate variation and notified cases of HFMD in selected cities of Shanxi Province, and to provide evidence for disease control and prevention. Meteorological variables and HFMD cases data in 4 major cities (Datong, Taiyuan, Changzhi and Yuncheng) of Shanxi province, China, were obtained from the China Meteorology Administration and China CDC respectively over the period 1 January 2009 to 31 December 2013. Correlations analyses and Seasonal Autoregressive Integrated Moving Average (SARIMA) models were used to identify and quantify the relationship between the meteorological variables and HFMD. HFMD incidence varied seasonally with the majority of cases in the 4 cities occurring from May to July. Temperatures could play important roles in the incidence of HFMD in these regions. The SARIMA models indicate that a 1° C rise in average, maximum and minimum temperatures may lead to a similar relative increase in the number of cases in the 4 cities. The lag times for the effects of temperatures were identified in Taiyuan, Changzhi and Yuncheng. The numbers of cases were positively associated with average and minimum temperatures at a lag of 1 week in Taiyuan, Changzhi and Yuncheng, and with maximum temperature at a lag of 2 weeks in Yuncheng. Positive association between the temperature and HFMD has been identified from the 4 cities in Shanxi Province, although the role of weather variables on the transmission of HFMD varied in the 4 cities. Relevant prevention measures and public health action are required to reduce future risks of climate change with consideration of local climatic conditions.

## Author Summary

Understanding of the impact of weather variables on HFMD transmission remains limited due to various local climatic conditions, socioeconomic status and demographic characteristics in different regions. This study provides quantitative evidence that the incidence of HFMD cases was significantly associated with temperature in Shanxi Province, North China. The delayed effects of weather variables on HFMD dictate different public health responses in 4 major cities in Shanxi Province. The results may provide a direction for local community and health authorities to perform public health actions, and the SARIMA models are helpful in the prediction of epidemics, determination of high-risk areas and susceptible populations, allocation of health resources, and the formulation of relevant prevention strategies. In order to reduce future risks of climatic variations on HFMD epidemics, similar studies in other geographical areas are needed, together with a longer study period to enable trend analysis which takes into consideration local weather conditions and demographic characteristics.

## Introduction

Hand, foot and mouth disease (HFMD) is an emerging infectious disease mainly caused by highly contagious intestinal viruses human enterovirus 71 (EV71) and coxsackievirus A16 (Cox A16) [1–3]. It is a human syndrome characterized by a distinct clinical presentation of fever, accompanied by oral ulcers and maculopapular rash or vesicular sores on the hands and feet, and sometimes the buttocks. HFMD transmission is through close personal contact, exposure to feces, contaminated objects and surfaces of an infected person. In recent decades, HFMD has become a growing public health threat to children, particularly those under the age of 5 [4–6]. Epidemics of HFMD are frequent and widespread in Asian countries, especially in China, Singapore, Malaysia and Japan, which have documented many large outbreaks of HFMD with severe complications and deaths predominantly among children [2,7–12]. At present, there is no specific curative treatment, and vaccine development is still in progress [13]. Weather variables might play a certain role in the transmission of the disease, as time series analysis in Guangzhou and Shenzhen, in China, showed that weather variation could affect the disease occurrence with a short lag period [14,15]. Under the context of global environmental change, the frequency of HFMD epidemics may be projected to increase in the future due to continued viral mutation, climate change, and the lack of health resources and effective surveillance systems in some regions [14,16–18]. Therefore, risk detection, early warning of HFMD cases with the capacity to predict a possible epidemic, and efficient public health response will be important to minimize the risk of epidemics and adverse impacts of HFMD.

Intestinal viruses have a worldwide distribution. In tropical and semitropical areas, they are present throughout the year, whereas in temperate climatic zones, they are more common during summer and fall [19]. A previous literature review has indicated that HFMD typically occurs in the summer and early autumn [20]. Such seasonal distribution suggests climatic variations may play a certain role in the transmission of the disease, particularly in temperate areas. The fifth assessment report of the Intergovernmental Panel on Climate Change (IPCC AR5) pointed out that the globe has experienced surface warming and projections of annual average temperature changes for 2081–2100 under Representative Concentration Pathways (RCPs) 2.6 and 8.5, relative to 1986–2005 [21]. In China, increases in temperature have been observed in most regions from south to north during the last decade [22]. Climate change has also been identified as an important risk factor for transmission of infectious diseases, especially vector and food borne diseases [23].

Since 2008, the China Ministry of Health has listed HFMD as a notifiable Class-C communicable disease, which has been included in the national communicable disease surveillance system and reporting network. Over six million cases had been reported up to the end of 2012 in China [17]. Studies have examined the association between HFMD and climate variables in selected regions [14,24–26]. Meteorological parameters, such as temperature and relative humidity, may affect the transmission and the frequency of HFMD. However, the effects of climate variables are not consistent in published studies, which could be due to various local climatic conditions, socioeconomic status and demographic characteristics in different regions. In particular, an understanding of the impact of seasonality and meteorological variables on disease transmission remains limited. A comparison among different cities within a Province may minimize potential confounding effects of socioeconomic inequalities and demographic differences, which will be used in this study to examine the role of climate variation on the incidence of HFMD, using Seasonal Autoregressive Integrated Moving Average (SARIMA) models.

The purpose of this study was to identify, with SARIMA models, the impact of meteorological variables on HFMD in 4 major cities of Shanxi in northern China, using existing surveillance data, and to quantify the relationship between climate variation and the incidence of HFMD. This study will provide scientific evidence to assist public health policy-making to carry out efficient prevention and control of HFMD.

## Materials and Methods

### Ethics statement

The study was approved by the Ethics Committee of Shanxi Medical University (No. 2013091), China, and conducted in accordance with its guidelines. Shanxi HFMD data were provided by the Shanxi Center for Disease Control and Prevention and were obtained from the National Surveillance System. No informed consent was required because no individual-level analysis was performed. The information contained in the patients’ records was anonymized and de-identified prior to analysis. Only aggregated data were analyzed and reported.

### Study areas

**Fig 1:**
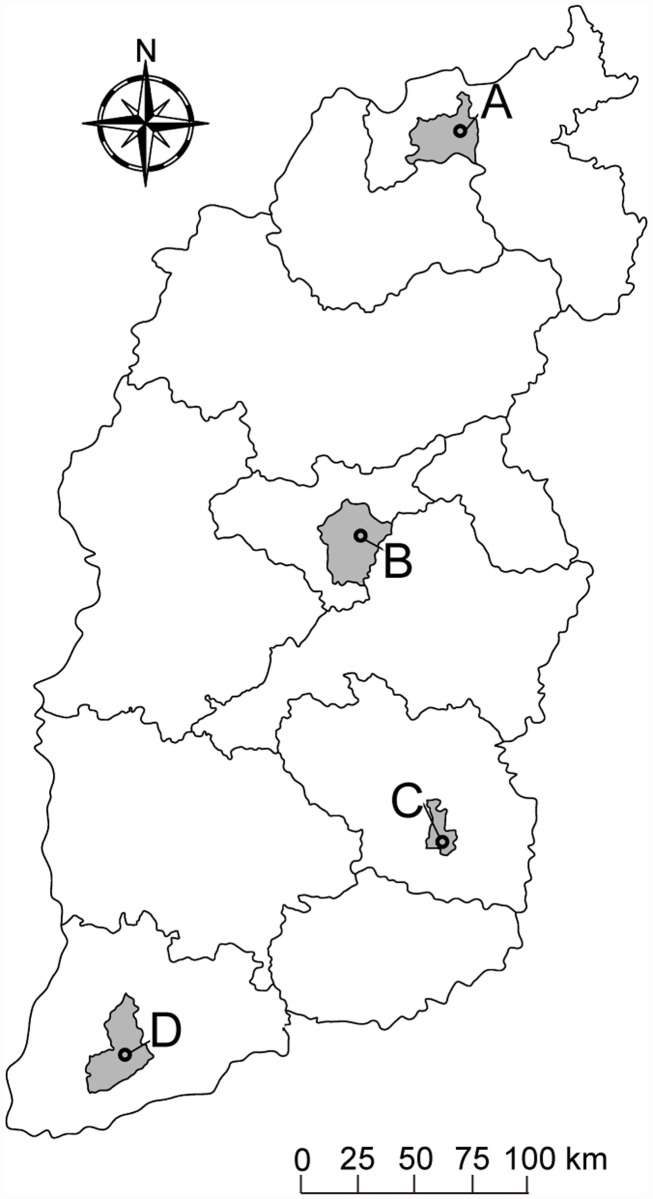
Locations of study areas in Shanxi, China. A, B, C, D: Datong, Taiyuan, Changzhi and Yuncheng, respectively.

Shanxi Province, which is located in North China, has a temperate, continental, monsoonal climate with four distinct seasons. The average temperature in January is in the range -16°C to -2°C and in July between 19°C and 28°C, the average rainfall is between 350 to 700 mm, and the average daily sunshine is between 7 to 9 hours. This study selected 4 major cities from north to south (Datong, Taiyuan, Changzhi and Yuncheng), which have similar socioeconomic and demographic conditions (Fig. 1). Datong (latitude 40°2′30" N and longitude 113°35′50" E) is the northernmost prefecture-level city of Shanxi Province, with a population of 3.36 million by the end of 2012 (data from the Shanxi Bureau of Statistics). Taiyuan (latitude 37°43' 36" N and longitude 112°28′14" E) is the capital and largest city of Shanxi, which is located at the centre of the province with an East-West span of 144 km and a North-South span of 107 km, and a population of 4.26 million in 2012. Changzhi (latitude 36°11′0" N and longitude 113°6′0" E) is the southeast city of Shanxi Province, with a population of 3.37 million in 2012. Yuncheng (latitude 35°1′33" N and longitude 111°0′19" E) is a southwestern city in Shanxi, with a population of 5.19 million in 2012.

### Data sources

Daily meteorological data including precipitation, average temperature, maximum temperature, minimum temperature, average relative humidity, and hours of sunshine for the study period from 1 January 2009 to 31 December 2013, were obtained from the Shanxi Meteorological Administration. Daily meteorological data were aggregated on a weekly basis which comprised a total period of 261 weeks.

Being a notifiable disease [17], all clinical and hospital doctors are required to report cases of HFMD to the local Center for Disease Control and Prevention. The diagnosis criteria for HFMD cases were provided in a guidebook published by the Chinese Ministry of Health [27,28]. Patients with HFMD have the following symptoms: fever, papules and herpetic lesions on the hands or feet, rashes on the buttocks or knees, inflammatory flushing around the rash and fluid in the blisters, or sparse herpetic lesions on the oral mucosa. A recent data quality survey report has demonstrated that the data are of high quality in China, with reporting completeness of 99.84% and accuracy of the information reported to be 92.76% [29]. In addition, in order to reduce apparent underreporting and a large number of missing information of patients from the early stage of the surveillance system in 2008, only the data from 2009 to 2013 were used for analysis. The weekly data of HFMD cases for the period were obtained from the China Information System for Disease Control and Prevention in Shanxi. According to our data, 90.9%, 91.0%, 90.3% and 97.8% HFMD cases were children aged 0–5 years in Datong, Taiyuan, Changzhi and Yuncheng, respectively. Therefore, we focused analysis on the incidence of HFMD among children aged 0–5 years in this study.

### Data analysis

The analysis includes descriptive, correlation and time series regression analyses. The meteorological variables data were calculated for intervals of 7 consecutive days, and transformed into a time series format. Descriptive analysis was performed by describing the distribution of climate variables and HFMD cases. Spearman rank correlation and partial correlation analysis were used to examine the association between each meteorological variable and the incidence of HFMD. In addition, given the potential lagged effect of the meteorological variables on disease transmission, cross-correlation analysis was also performed with relevant time lag values. Time series analysis was used to assess the effect of climatic variables on HFMD incidence.

**Fig 2:**
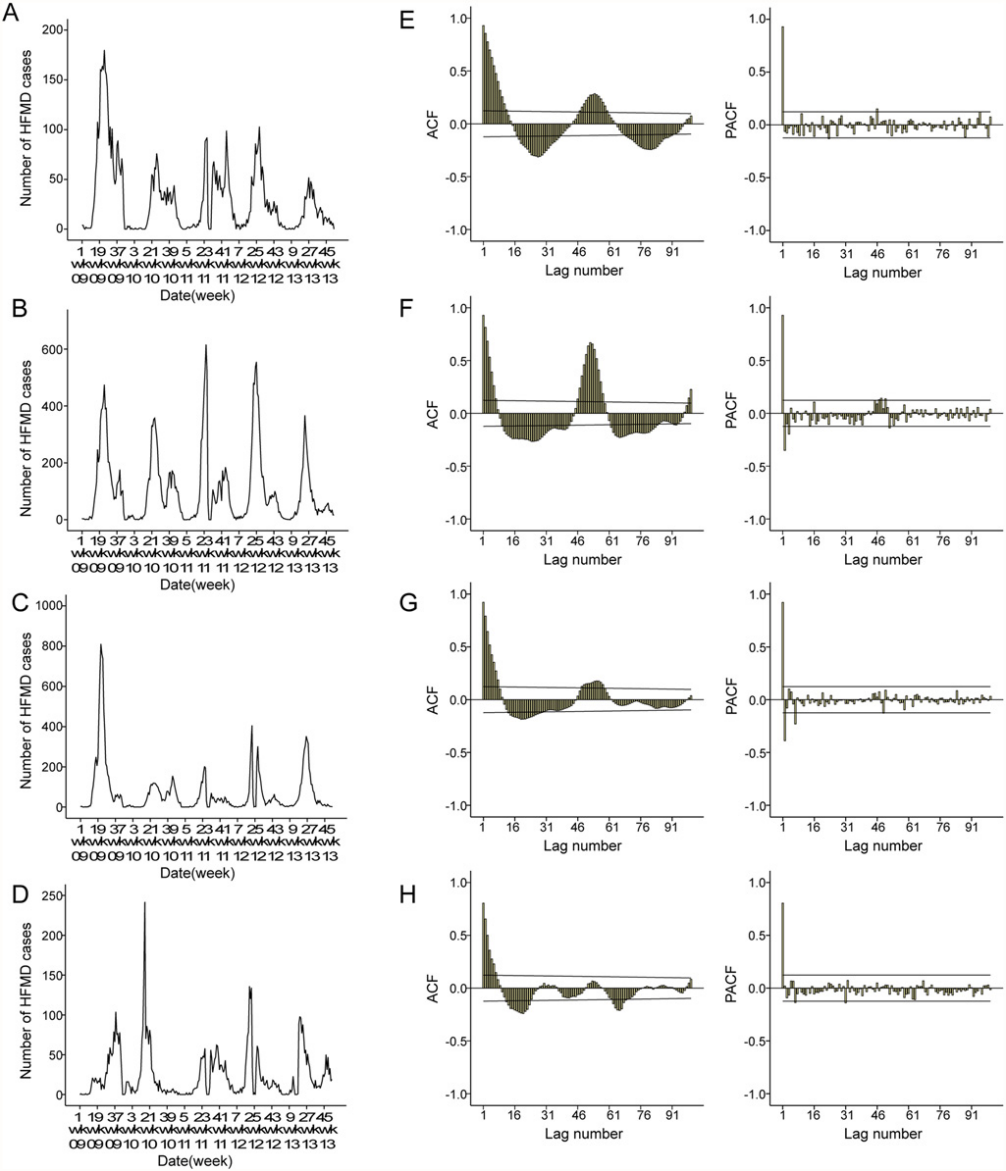
Notified weekly cases of hand-foot-mouth disease (HFMD) and autocorrelation function (ACF) and Partial ACF (PACF) plot of original number of HFMD cases in 4 cities, Shanxi, 2009–2013. A and E: the number of HFMD cases, ACF and PACF plots of Datong. B and F: the number of HFMD cases, ACF and PACF plots of Taiyuan. C and G: the number of HFMD cases, ACF and PACF plots of Changzhi. D and H: the number of HFMD cases, ACF and PACF plots of Yuncheng.

The plot of the observed HFMD incidence showed most of the cases occurred from May to July in the 4 cities (Fig. 2A-2D). Furthermore, the plots of autocorrelation function (ACF) and partial auto correlation function (PACF) of HFMD cases (Fig. 2E-2H) showed the time series was non-stationary. With the temporal dependence of HFMD incidence, the need to use a SARIMA model was evident. The Seasonal Autoregressive Integrated Moving Average (SARIMA) model (Box and Jenkins method) has been recently applied in epidemiological studies [24,30–33], and was used to describe current (and future) incidence of HFMD in terms of their past values in this study. SARIMA models extend basic ARIMA models and allow for the incorporation of seasonal patterns. A SARIMA model, which includes seasonal and non-seasonal components, is typically represented by (p,d,q)(P,D,Q)s, where p represents the order of autoregression (AR), d is the order of differencing, and q is the order of the moving average (MA). P, D, and Q are their seasonal counterparts, and s is the seasonal lag [34]. The long-term trend and seasonal components of each time series can be removed using SARIMA models [24]. In addition, we considered the weather during holidays may also affect the occurrence of HFMD.

**Fig 3:**
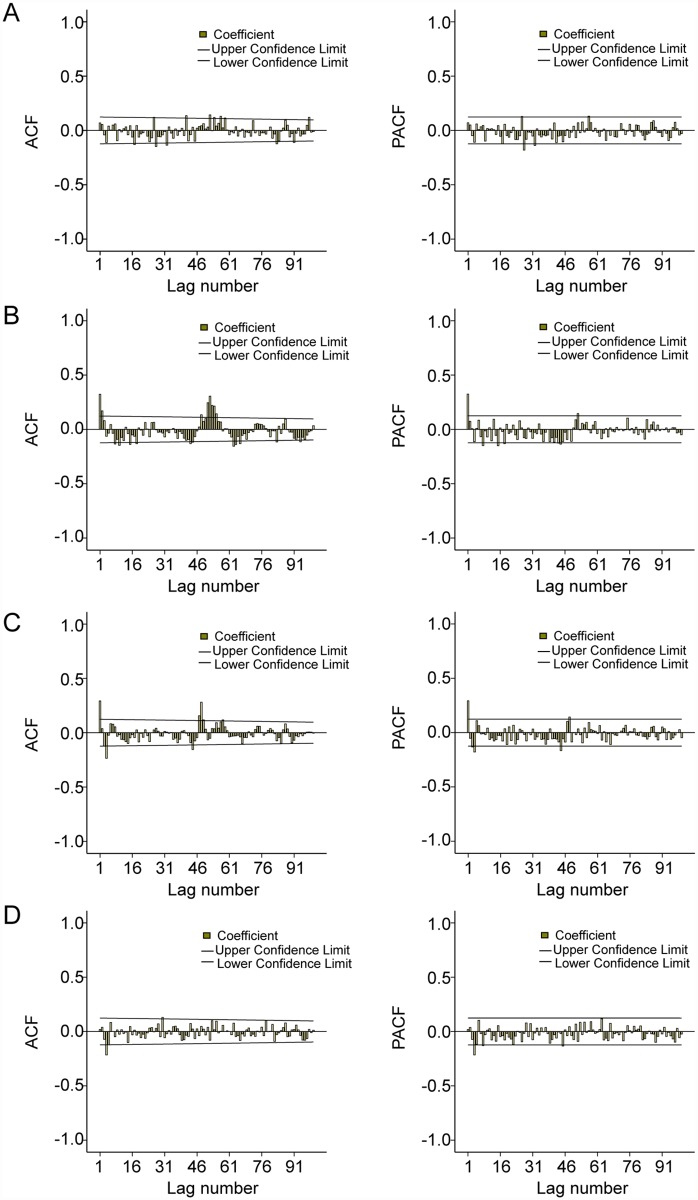
ACF and PACF plots of integrated the number of HFMD cases. A, B, C and D show ACF and PACF plots for Datong, Taiyuan, Changzhi and Yuncheng.

The development of a SARIMA model is a four-step process. Therefore, for the HFMD time series analysis, it was firstly necessary to stabilize the variance of the series by square root transformation, and seasonal and regular differencing was also applied. Secondly, in order to identify the order of MA and AR parameters, the structure of temporal dependence of stationary time series was assessed respectively, by the analysis of autocorrelation (ACF) and partial autocorrelation (PACF) functions. From the correlograms of the series, the p value may equal 0, 1 or 2 for autoregressive parameters and q value may equal 1, 2 or 3 for moving average parameters (Fig. 3A-3D). Thirdly, parameters of the model were estimated by using the maximum likelihood method. The goodness-of-fit of the models was determined for the most appropriate model (the lowest normalized Bayesian Information Criteria (BIC) and the highest stationary R square (R^2^)), using the Ljung-Box test that measures both ACF and PACF of the residuals, which must be equivalent to white noise. The significance of the parameters should be statistically different from zero. Finally, the predictions were performed by using the best fitting model. The predictive validity of the models was evaluated by calculating the root mean square error (RMSE), which measures the amount by which the fitted values differ from the observed values. The smaller the RMSE, the better the model is for forecasting. Therefore, the SARIMA model was developed and verified by dividing the data file into two date sets: the data from the 1^st^ calendar week of 2009 to the 52^nd^ calendar week of 2012 were used to construct a model; and those from the 1^st^ calendar week to the 52^nd^ calendar week of 2013 were used to validate it. For statistical analysis SPSS version 19.0 and Stata version 12.0 were used.

The sensitivity analysis was conducted based on daily unit to check whether the number of weekly HFMD cases could affect the result estimates. Meanwhile, we controlled for day of the week and public holidays using categorical indicator variables. In addition, we graphically examined the exposure-response curves derived using a smoothing function [28,35–37], and natural cubic splines [35] to control long-term trend and seasonality with 6 df per year for time [37], which was done using the distributed lag non-linear models (dlnm) package in the software R.

## Results

### Descriptive analysis

Meteorological variables and number of HFMD cases show differences from the northern city to the southern city (Table 1). The northern city (Datong) has a lower temperature and relative humidity than the central city (Taiyuan) and southern cities (Changzhi and Yuncheng); while the southern cities have less sunshine than the central city and northern city. During the study period, the number of HFMD cases in Taiyuan and Changzhi were more than that in Datong and Yuncheng.

**Table 1:** Meteorological variables and number of HFMD cases in 4 cities, Shanxi, 2009–2013.

Variable	City	Min	5% centile	Median	95% centile	Max	Mean
Weekly mean precipitation (mm)	Datong	0.0	0.0	2.0	934.6	1868.6	244.5
	Taiyuan	0.0	0.0	1.1	936.9	1409.2	253.7
	Changzhi	0.0	0.0	4.3	938.3	2335.7	287.9
	Yuncheng	0.0	0.0	1.4	934.3	1402.6	173.2
Weekly average temperature (°C)	Datong	-17.0	-12.4	8.7	23.6	28.1	7.4
	Taiyuan	-10.0	-6.4	11.9	25.3	28.2	11.0
	Changzhi	-10.6	-6.7	11.8	23.4	25.4	10.0
	Yuncheng	-4.7	-3.2	15.5	28.7	30.7	14.3
Weekly mean maximum temperature (°C)	Datong	-9.2	-5.9	16.0	30.	34.6	14.4
	Taiyuan	-3.8	-2.1	19.6	31.9	34.7	17.6
	Changzhi	-4.6	5.3	18.9	30.0	32.7	16.7
	Yuncheng	-2.3	3.6	22.1	34.7	48.2	22.1
Weekly mean minimum temperature (°C)	Datong	-23.9	-17.7	2.4	18.2	22.2	1.2
	Taiyuan	-16.2	-10.9	6.3	20.2	22.9	5.6
	Changzhi	-15.5	-12.0	5.6	18.5	21.4	4.6
	Yuncheng	-10.6	-7.6	10.3	24.2	26.3	9.4
Weekly average relative humidity (%)	Datong	21.4	27.8	52.0	75.1	83.7	51.8
	Taiyuan	20.6	29.6	53.6	76.3	84.6	53.1
	Changzhi	22.6	33.7	59.7	84.4	91.3	60.1
	Yuncheng	25.4	34.3	56.0	76.2	87.4	56.1
Weekly mean hours of sunshine (%)	Datong	1.6	4.2	7.5	10.1	11.4	7.4
	Taiyuan	0.9	2.6	7.0	10.7	11.5	6.8
	Changzhi	0.7	2.7	6.2	9.6	11.0	6.3
	Yuncheng	0.0	1.5	5.4	9.2	11.6	5.4
Weekly number of HFMD cases	Datong	0	0	17	103	180	30
	Taiyuan	0	0	60	392	616	105
	Changzhi	0	0	29	303	811	70
	Yuncheng	0	0	13	81	242	24

### Correlation analysis

In the 4 study cities, precipitation, temperature, relative humidity and hours of sunshine were positively correlated with incidence of HFMD (*p* < 0.05). Different meteorological variables may also be correlated with each other. For example, average temperature was positively correlated with maximum temperature in the 4 cities from north to south (*r*_s_ = 0.994, 0.990, 0.990, 0.981; *p* < 0.001, respectively), and also correlated with minimum temperature (*r*_s_ = 0.993, 0.988, 0.987, 0.989; *p* < 0.001, respectively). Accounting for these correlations, the association between meteorological variables and the number of HFMD cases were then analyzed using partial correlations. Results showed the associations of increased number of HFMD cases with increasing atmospheric temperature in the 4 cities (*p* < 0.05). In addition, the results showed statistically significant but weaker correlation for the association between relative humidity and the incidence of HFMD in Taiyuan, as well as the weaker correlation between precipitation and HFMD in Changzhi (Table 2).

**Table 2:** Spearman rank correlations and partial correlations between weekly meteorological variables and HFMD.

Variable	City	Spearman rank correlation		Partial correlation	
		*r*_s_	*p*	*r*_s_	*p*
Precipitation (mm)	Datong	0.282	<0.001	0.109	0.080
	Taiyuan	0.352	<0.001	0.103	0.100
	Changzhi	0.214	0.001	0.143	0.022
	Yuncheng	0.184	0.003	0.067	0.282
Average temperature (°C)	Datong	0.610	<0.001	0.513	<0.001
	Taiyuan	0.726	<0.001	0.519	<0.001
	Changzhi	0.715	<0.001	0.381	<0.001
	Yuncheng	0.462	<0.001	0.265	<0.001
Average max temperature (°C)	Datong	0.596	<0.001	0.493	<0.001
	Taiyuan	0.721	<0.001	0.502	<0.001
	Changzhi	0.707	<0.001	0.366	<0.001
	Yuncheng	0.454	<0.001	0.158	0.011
Average min temperature (°C)	Datong	0.737	<0.001	0.528	<0.001
	Taiyuan	0.707	<0.001	0.511	<0.001
	Changzhi	0.707	<0.001	0.381	<0.001
	Yuncheng	0.475	<0.001	0.258	<0.001
Average relative humidity (%)	Datong	0.181	0.003	-0.071	0.257
	Taiyuan	0.158	0.011	-0.180	0.004
	Changzhi	0.209	0.001	-0.095	0.127
	Yuncheng	0.193	0.002	0.058	0.355
Hours of sunshine (h)	Datong	0.331	<0.001	0.025	0.688
	Taiyuan	0.322	<0.001	-0.064	0.308
	Changzhi	0.234	<0.001	0.070	0.262
	Yuncheng	0.155	0.012	0.052	0.406

### Time series analysis

**Fig 4:**
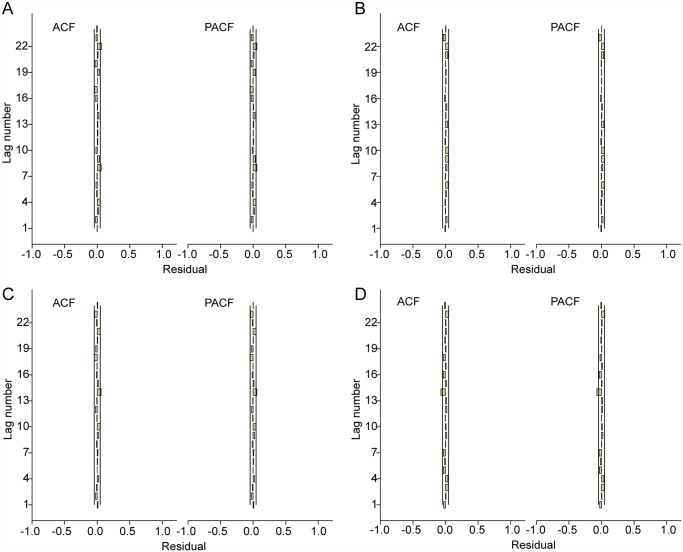
ACF and PACF plots of residuals after applying the appropriate SARIMA model. A: ACF and PACF plot of residuals of SARIMA (0,1,1)(2,0,1)_52_ model for Datong. B: ACF and PACF plot of residuals of SARIMA (2,1,3)(1,1,1)_52_ model for Taiyuan. C: ACF and PACF plot of residuals of SARIMA (0,1,1)(0,1,1)_52_ model for Changzhi. D: ACF and PACF plot of residuals of SARIMA (0,1,1)(1,1,2)_52_ model for Yuncheng.

In order to estimate the values of parameters in fitted models, these models were diagnosed by analyzing the data with several SARIMA models without the weather variables, and the models in which the residual was not likely to be white noise were excluded. Therefore, the univariate SARIMA (0,1,1)(2,0,1)_52_ model for Datong; SARIMA (2,1,3)(1,1,1)_52_ model for Taiyuan; SARIMA (0,1,1)(0,1,1)_52_ model for Changzhi; and SARIMA (0,1,1)(1,1,2)_52_ model for Yuncheng had both the lowest Bayesian information criterion (BIC) and the highest R^2^ values and were the best to fit the HFMD cases, respectively (Table 3). The Ljung-Box test confirmed that the residuals of the time series were not statistically dependent (*p* > 0.05) and the residuals on ACF and PACF plots showed the absence of persistent temporal correlation (Table 3) (Fig. 4). The selected SARIMA model fitted the observed data from 2009 to 2012. Then, the model was used to project the number of HFMD cases between January to December 2013, and was validated by the actual observations. The validation analysis suggested that the model had reasonable accuracy over the predictive period in the 4 cities (root-mean-square error (RMSE) = 1.763, 4.505, 4.907 and 2.817, respectively) (Table 3).

**Table 3:** Characteristics of SARIMA models for HFMD in four cities.

City	SARIMA model	Statistic parameters										
	(p,d,q)(P,D,Q)s	AR2	MA1	MA3	SAR1	SAR2	SMA1	SMA2	*R* ^*2*^	BIC	*p*	RMSE
Datong	(0,1,1)(2,0,1)_52_	-	0.722	-	-	-0.090	0.985	-	0.361	2.225	0.273	1.763
Taiyuan	(2,1,3)(1,1,1)_52_	-0.878	-	0.481	0.078	-	0.997	-	0.587	3.824	0.060	4.505
Changzhi	(0,1,1)(0,1,1)_52_	-	0.474	-	-	-	0.999	-	0.584	3.499	0.537	4.907
Yuncheng	(0,1,1)(1,1,2)_52_	-	0.664	-	-0.719	-	-	0.748	0.644	2.039	0.310	2.817

SARIMA: Seasonal Autoregressive Integrated Moving Average model,

AR: autoregressive,

MA: moving average,

SAR: seasonal autoregressive,

R^2^: Stationary R-squared,

BIC: Bayesian information criteria,

*p*: Ljung-Box test,

RMSE: Root Mean Square Error.

The cross-correlation analyses showed the lag effects of the meteorological variables on the number of HFMD cases were different in the 4 cities. In Datong, HFMD was significantly positively associated with average temperature at lag 0 (coefficients = 0.540, *p* < 0.05), mean maximum temperature at lag 0 (coefficients = 0.520, *p* < 0.05), mean minimum temperature at lag 0 (coefficients = 0.542, *p* < 0.05). In Taiyuan, the cases were significantly positively associated with average temperature at lag 1 week (coefficients = 0.615, *p* < 0.05), maximum temperature at lag 1 week (coefficients = 0.612, *p* < 0.05), minimum temperature at lag 1 week (coefficients = 0.606, *p* < 0.05), relative humidity at lag 3 weeks (coefficients = 0.224, *p* < 0.05). In Changzhi, the disease was significantly positively associated with average temperature, maximum and minimum temperature at lag 1 week (coefficients = 0.479, 0.472 and 0.465, *p* < 0.05), respectively. In Yuncheng, HFMD was significantly positively associated with average temperature at lag 1 week (coefficients = 0.351, *p* < 0.05), maximum temperature at lag 2 weeks (coefficients = 0.347, *p* < 0.05) and minimum temperature at lag 1 week (coefficients = 0.352, *p* < 0.05).

To reduce potential multicollinearity, weekly average temperature, weekly mean maximum temperature and weekly minimum temperature were put into separate regression models (Models 1, 2 and 3). In Model 1, 2 and 3, temperature (average, maximum, and minimum, respectively) was included, along with other meteorological variables. The results indicated that average temperature, and maximum and minimum temperatures with different lag times were significant in the SARIMA Model 1, Model 2 and Model 3, respectively (Table 4). Overall, SARIMA models with temperature were a better fit and validity than the models without the variable (Stationary R-squared (Stationary R^2^) increased, while the BIC decreased) (Table 3 and Table 4). Other meteorological variables were not significantly included in the models, indicating their contribution was not statistically significant in this study.

**Table 4:** Characteristics of SARIMA models with climate variables for HFMD in four cities.

City	Coefficient of parameters									*R* ^*2*^	BIC	*p*	95% CI	RMSE
	AR1	AR2	MA1	MA2	SAR1	SMA1	Lag 0	Lag 1	Lag 2					
Model 1 with average temperature														
Datong	-	-	-	-	-0.163	-	0.008	-	-	0.456	1.148	0.002	(0.003, 0.012)	1.759
Taiyuan	-	-0.716	-	-0.294	-	0.953	-	0.014	-	0.593	2.986	0.024	(0.005, 0.027)	4.378
Changzhi	0.303	-	-	-	-	-0.365	-	0.011	-	0.631	3.088	0.042	(0.001, 0.021)	4.571
Yuncheng	0.026	-	-	-	—0.525	-0.741	-	0.021	-	0.692	1.995	0.028	(0.004, 0.047)	2.650
Model 2 with maximum temperature														
Datong	-	-	-	-	-0.161	-	0.006	-	-	0.379	2.221	0.009	(0.001, 0.010)	1.745
Taiyuan	-	-0.305	-	-0.451	0.431	-	-	0.010	-	0.590	3.818	0.009	(0.003, 0.018)	4.397
Changzhi	0.356	-	-	-	-	-0.381	-	0.016	-	0.629	3.450	0.006	(0.004, 0.027)	4.826
Yuncheng	-	-	-	-	-	0.264	-	-	0.014	0.659	1.967	0.032	(0.003, 0.084)	2.088
Model 3 with minimum temperature														
Datong	-	-	0.095	-	-0.242	0.095	0.007	-	-	0.379	2.228	0.007	(0.002, 0.013)	1.741
Taiyuan	0.430	-	-	-	-0.556	-	-	0.011	-	0.624	3.133	0.019	(0.002, 0.021)	4.374
Changzhi	-	-	0.357	-	-	-0.276	-	0.015	-	0.586	3.231	0.016	(0.003, 0.027)	4.750
Yuncheng	-	-	-	0.027	-	0.276	-	0.019	-	0.689	1.893	0.007	(0.001, 0.058)	2.170

AR2: 2-order autoregression;

MA1, 2: 1, 2-order moving average;

SAR1: 1-order seasonal autoregression;

SMA1: 1-order seasonal moving average;

Lag 0, 1, 2: 0, 1, 2 weeks prior.

**Fig 5:**
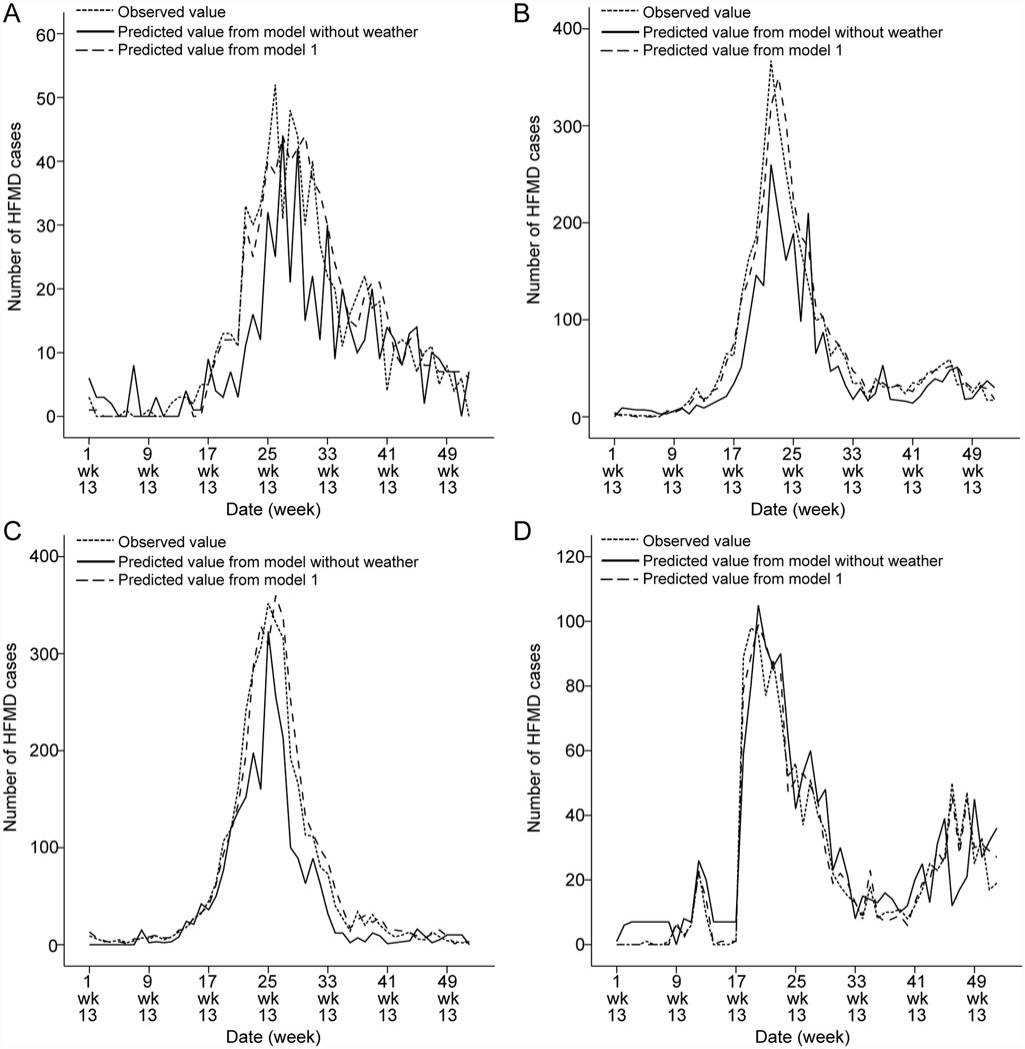
Prediction of the number of HFMD cases on the basis of the model without and with weather variables. A, B, C, D: Datong, Taiyuan, Changzhi and Yuncheng, respectively.

The models suggest that in Datong, a 1°C rise in weekly average temperature, weekly mean maximum temperature and weekly mean minimum temperature may be related to an increase in the weekly number of cases of HFMD of 0.8% (95%CI: 0.3%-1.2%), 0.6% (95%CI: 0.1%-1.0%) and 0.7% (95%CI: 0.2%-1.3%) respectively. In Taiyuan, a 1°C rise in weekly average temperature, weekly mean maximum temperature and weekly mean minimum temperature may be related to an increase in the weekly number of cases of HFMD of 1.4% (95%CI: 0.5%-2.7%), 1.0% (95%CI: 0.3%-1.8%) and 1.1% (95%CI: 0.2%-2.1%) respectively. In Changzhi, a 1°C rise in weekly average temperature, weekly mean maximum temperature and weekly mean minimum temperature may be related to an increase in the weekly number of cases of HFMD of 1.1% (95%CI: 0.1%-2.1%), 1.6% (95%CI: 0.4%-2.7%) and 1.5% (95%CI: 0.3%-2.7%) respectively. Finally, in Yuncheng a 1°C rise in weekly average temperature, weekly mean maximum temperature and weekly mean minimum temperature may be associated with an increase in the weekly number of cases of HFMD of 2.1% (95%CI: 0.4%-4.7%), 1.4% (95%CI: 0.3%-8.4%) and 1.9% (95%CI: 0.1%-5.8%) respectively (Table 4). Table 4 indicates that the incidence of the disease may rise with the increase of temperature, but only within a certain range of temperatures. The selected SARIMA model was used to project the number of HFMD cases in each city for the 52 weeks between January and December 2013. The validation for January to December 2013 data showed a good fit between observed and predicted data (Fig. 5).

A comparison between the models for the 4 cities, reveals that although a 1°C rise in temperature may cause a similar relative increase in the number of cases, the lag times for the effects of temperatures were shorter in Datong (at lag 0) than those in Taiyuan, Changzhi and Yuncheng (at lag 1 week). The lag times for the effects of maximum temperature were longer in Yuncheng (at lag 2 weeks) than those in Changzhi, Taiyuan and Datong (Table 4).

**Fig 6:**
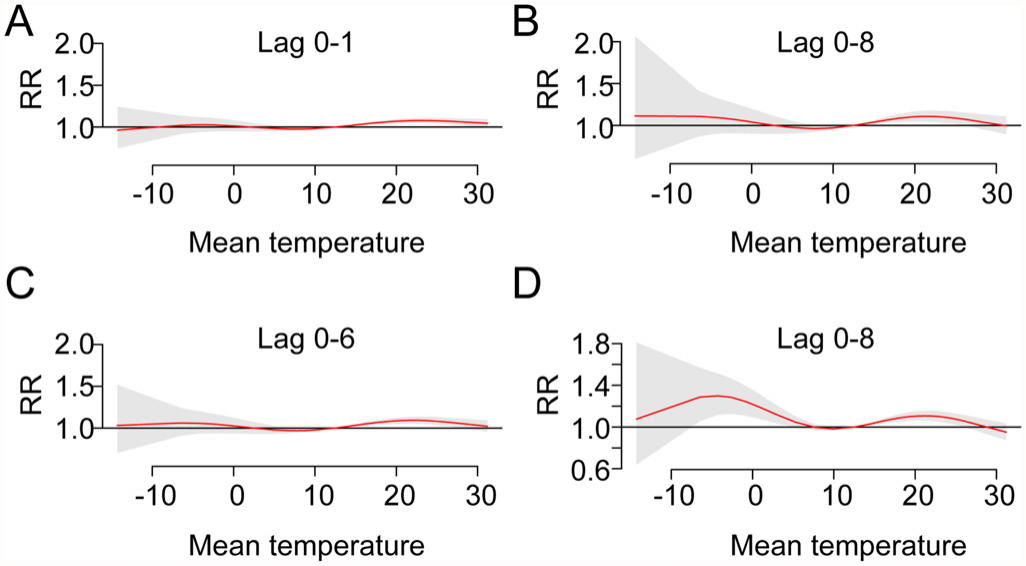
Dose-response curves of temperature with HFMD occurrence in 4 cities along lags. The Y axes represent the relative risk (RR) for HFMD within a certain range of temperatures; the grey-shaded areas are 95% CIs. A, B, C, D: Datong, Taiyuan, Changzhi and Yuncheng, respectively.

In the sensitivity analysis, the results using daily data indicated that a 1°C rise in daily average temperature may be related to an increase in the daily number of cases of HFMD of 1.2% (95%CI: 0.1%-2.3%) at lag 1 day, 1.6% (95%CI: 1.0%-2.2%) at lag 8 days, 1.5% (95%CI: 0.5%-2.5%) at lag 6 days, and 2.4% (95%CI: 0.1%-4.7%) at lag 8 days in Datong, Taiyuan, Changzhi and Yuncheng, respectively. Although the analysis of daily data is likely to yield a more precise estimate compared to weekly data, the results were similar. Fig. 6 shows non-linear dose-response relationships for temperature with HFMD occurrence in the 4 cities, and an increase in HFMD occurrence within a short interval.

## Discussion

HFMD has become an important public health concern in the affected countries and has attracted an increasing research interest [38–40]. In China, HFMD has been a notifiable infectious disease since 2008 and appropriate prediction of risk may aid in the effective control of the disease nationwide. Despite the evidence that shows that climate variation is closely linked with a huge burden of vector and food borne diseases at the global scale, the role of weather variables in HFMD transmission is still not well understood. This is because its epidemiological characteristics may vary over different geographic locations due to various climatic and socioeconomic situations in China. This study is the first to compare the association between climatic variables and the HFMD transmission among different cities within a Province. The results suggest that temperature may play an important role in the transmission of HFMD in the 4 cities although the lag times for the effects of temperatures differed. This may aid in the projection of the disease and in disease control and prevention, especially in an era of global environmental change.

HFMD has a seasonal distribution, and there are different epidemiological features in different regions due to various climatic, geographic and socioeconomic factors. In Singapore, there is a large peak from mid-March to the end of May and a smaller peak from early October to early December 2008 [41]. In Japan, one single peak was detected in July 2011, particularly in western Japan [12]. In Taiwan, a study showed HFMD peaked in June and disappeared after August 2008 [42]. In Hong Kong, a seasonal peak was detected in the warmer months (May-July), along with a smaller winter peak (October-December) from 2001 to 2009 [43].

In mainland China, the number of reported cases of HFMD and its associated morbidity and mortality varies remarkably among various Provinces. The seasonal peaks can vary from a single peak to double peaks in some Provinces (such as Henan, Shandong, Guangdong) [24,44,45]. There is also a trend for the disease to move from the south to the north between May and June [46]. In this study, HFMD incidence varied seasonally with the majority of cases in the 4 cities occurring from May to July, which is typically one month later than that observed in other Provinces [45]. Specifically, the seasonal patterns of HFMD epidemics were different in the 4 cities. In the central city of Taiyuan, two peaks per year were observed in the warmer months and winter months, and the highest peak was present from May to July, accompanied by a smaller peak from October to December. However, in the northern city and the southern cities, HFMD presented once per year and biannual patterns were evident. The cases peaked in 2009, 2009 and 2010 in Datong, Changzhi and Yuncheng, respectively, indicating HFMD cases occur in spatio-temporal clusters [17]. The SARIMA models also showed the seasonal variation in the 4 cities. These findings are similar to those of other studies investigating the epidemiological features of HFMD [47,48].

Global climate projections have suggested an increase in the distribution and prevalence of infectious diseases in association with climate change [49]. Climate variations may change the reproductive capacity of infectious pathogens and vectors, alter the survival of viruses in the physical environment, alter patterns of water and food use, change human behaviour, and increase disease prevalence [50,51]. It was observed from this study that HFMD incidence was significantly associated with meteorological variables, which is consistent with previous studies [7,14,24–26]. However, previous studies have not excluded the potential confounding factor of socioeconomic status, and findings have been inconsistent. In this study, we compared the time series data within Shanxi Province, using 4 major cities with similar socioeconomic status. Our results indicated that temperature could be the key climatic indicator in the transmission of HFMD. Moreover, the SARIMA models suggested that although the pattern differed in each city, temperature had a significant impact on the transmission of HFMD in the 4 cities. The SARIMA models, which allow the integration of external factors (e.g. climatic variables), are a useful tool for interpreting and applying surveillance data in disease control and prevention [24]. Our study shows that, within a certain range of temperature variation, a 1°C rise in average temperature may lead to 0.8%, 1.4%, 1.1% and 2.1% increase in the number of cases of HFMD in Datong, Taiyuan, Changzhi and Yuncheng, respectively. This result is similar to other research on the effects of temperature on enteric infectious diseases [25,31,52]. In addition, the results show that a 1°C rise in maximum and minimum temperatures may also be related to increases in the number of cases in these regions, indicating temperature could be used as a forecasting factor in public health practice.

Although the associations between HFMD and other climate variables including precipitation, average relative humidity and hours of sunshine are statistically significant in the correlation analyses, they were not significant in the SARIMA models. In the context of global warming, precipitation, relative humidity and hours of sunshine may contribute to the transmission of enteric infectious diseases by affecting the ecological environment of pathogens, exposure probability and host susceptibility, thus maybe resulting in the incidence of diseases [49]. Therefore, further studies are necessary to better understand the role of these climatic factors on HFMD.

The time lag effect refers to the delay between the time of an exposure and the subsequent development of a disease. The time lag effects of temperature were observed in the central and southern cities of Taiyuan, Changzhi and Yuncheng, but no lag effect was detected in the northern city of Datong. These findings suggest HFMD cases may be forecast 1 week ahead in Taiyuan, Changzhi and Yuncheng according to weekly average temperature and minimum temperature, and 2 weeks ahead in Yuncheng according to weekly maximum temperature. This result is similar to a previous study conducted in Japan investigating the relationship between temperature and HFMD, with lag period 0–3 weeks [25]. Other studies showed the role of temperature and relative humidity at 2 weeks lag time in Hong Kong [26], and the role of temperature and relative humidity at 1 week lag time in Guangzhou [14]. On the contrary, our study has not detected the role of relative humidity on HFMD. These various impacts of climatic variables on HFMD could be attributed to local climate conditions and other socioeconomic characteristics. Therefore, local climate factors, together with other variables should be carefully considered for public health professionals to prevent or reduce future risks of HFMD incidence. Due to the different lag time effect in these cities, the response from disease surveillance systems should be different.

The limitations of this study should be acknowledged. Firstly, weekly data rather than daily data may underestimate the relationship between climate variables and HFMD incidence. This may affect the accuracy of exposure assessment. Secondly, the Seasonal Autoregressive Integrated Moving Average (SARIMA) model has been recently applied in modeling and projecting for both non-linear and non-stationary time series, but other functions are required to assess the dose-response curve for temperature and HFMD occurrence. Thirdly, we were unable to differentiate the pathogens of HFMD cases reported to CDC surveillance system. Therefore, it was not possible to examine the specific impacts of climatic conditions on different pathogens. In addition, our analysis is exploratory, and we are unable to exclude the possibility of a spurious finding or unmeasured confounding factors that may be associated with both weather/ecological variables and HFMD occurrence, which has been a common challenge for ecological studies. Although we considered that holidays may affect the occurrence of HFMD, it was difficult to define a holiday variable using weekly data, and it was not possible to combine holidays (in summer and winter). Despite these shortcomings, these findings are useful as they provide information to better understand the effect of climate variation on HFMD, and this study may inform policy makers in the development of efficient prevention strategies.

### Conclusion

Although there are some time series studies in China on HFMD, these studies scarcely considered the potential impact of socioeconomic status. The findings in this study indicate that the occurrences of HFMD were positively associated with temperature in 4 major cities which have similar socioeconomic status and demographic characteristics. In addition, different lag effects of temperature were observed in selected regions from north to south. The results will be useful to assist public health responses in the different regions and informing local community and health authorities to better predict disease outbreaks.
